# Candidate SNP Markers of Chronopathologies Are Predicted by a Significant Change in the Affinity of TATA-Binding Protein for Human Gene Promoters

**DOI:** 10.1155/2016/8642703

**Published:** 2016-08-22

**Authors:** Petr Ponomarenko, Dmitry Rasskazov, Valentin Suslov, Ekaterina Sharypova, Ludmila Savinkova, Olga Podkolodnaya, Nikolay L. Podkolodny, Natalya N. Tverdokhleb, Irina Chadaeva, Mikhail Ponomarenko, Nikolay Kolchanov

**Affiliations:** ^1^Children's Hospital Los Angeles, University of Southern California, Los Angeles, CA 90027, USA; ^2^Institute of Cytology and Genetics, Siberian Branch of Russian Academy of Sciences, Novosibirsk 630090, Russia; ^3^Department of Natural Sciences, Novosibirsk State University, Novosibirsk 630090, Russia

## Abstract

Variations in human genome (e.g., single nucleotide polymorphisms, SNPs) may be associated with hereditary diseases, their complications, comorbidities, and drug responses. Using Web service SNP_TATA_Comparator presented in our previous paper, here we analyzed immediate surroundings of known SNP markers of diseases and identified several candidate SNP markers that can significantly change the affinity of TATA-binding protein for human gene promoters, with circadian consequences. For example, rs572527200 may be related to asthma, where symptoms are circadian (worse at night), and rs367732974 may be associated with heart attacks that are characterized by a circadian preference (early morning). By the same method, we analyzed the 90 bp proximal promoter region of each protein-coding transcript of each human gene of the circadian clock core. This analysis yielded 53 candidate SNP markers, such as rs181985043 (susceptibility to acute Q fever in male patients), rs192518038 (higher risk of a heart attack in patients with diabetes), and rs374778785 (emphysema and lung cancer in smokers). If they are properly validated according to clinical standards, these candidate SNP markers may turn out to be useful for physicians (to select optimal treatment for each patient) and for the general population (to choose a lifestyle preventing possible circadian complications of diseases).

## 1. Introduction

Diurnal (circadian) oscillations of the expression level have been reliably identified in ~10000 genes of placental mammals [1]. The circadian clock of mammals is a system of self-sustained oscillators that function under the control of a central circadian pacemaker located in suprachiasmatic nuclei of the hypothalamus [2]. They synchronize all processes in living organisms, from gene transcription to behavior, thus ensuring their temporal adaptation to 24-hour days on Earth [1]. The minimal set of 12 genes—*CLOCK*,* ARNTL*,* ARNTL2*,* PER1*,* PER2*,* CRY1*,* CRY2*,* CSNK1E*,* CSNK1D*,* RORΑ*,* RORС*, and* NR1D1*—forms the core of the molecular genetic mechanism of the circadian clock, whose functioning is based on feedback relations among its components [3, 4] and on the relations of these genes with the entry points for external signals, which modulate parameters of the circadian clock in response to such external stimuli as light and food [5]. Via the retinohypothalamic tract, the central circadian oscillator imposes a rhythm on peripheral oscillators, which share their molecular genetic structure but work in each cell in accordance with their own specific rhythms of organs, tissues, and systems of tissues [1]. All these oscillators set the rhythm for a multitude of genes via expression of tissue-specific transcription factors (short-term regulation) or chromatin remodeling (long-term regulation) [6, 7]. Indeed, transcriptomic studies have shown that genes that are subject to circadian control are characterized by overrepresentation of short GC-rich and TA-rich motifs for binding of transcription factors (e.g., TBP-binding motifs) [8, 9] in comparison with genome-wide average values of these parameters. In addition, an empirical study [10] revealed that CLOCK-ARNTL is a pioneer-like transcription factor that interacts with nucleosomes for rhythmic chromatin opening. Adjustment of the peripheral oscillation to the general circadian rhythm synchronizes the functioning of various systems of organs, whereas their desynchronosis can worsen or cause pathological changes in systems that are not interacting directly (e.g., autoimmune disorders may be caused by desynchronosis of the immune defense of the body from exotoxins and excretory/metabolizing systems dealing with analogous endotoxins [11]). Chronopharmacology is concerned with identification of circadian optima for diagnosis [12] and treatment [13, 14].

Experiments on genetic animal models have shown that, in addition to changes in parameters of the circadian clock (amplitude, a phasic response to external signals, or the period of free-flowing rhythm), the mutant animals develop such disorders as metabolic syndrome; disturbances in the system of gluconeogenesis or lipogenesis, in renal function, or in thermogenesis; and development of tumors [15, 16]. Furthermore, research in the field of genetic epidemiology uncovered associations of single nucleotide polymorphisms (SNPs) of circadian clock genes with a wide range of pathological states [17, 18]. A large number of such SNPs are located in noncoding regions of genes (these regions are responsible for regulation of expression). Functional annotation of regulatory SNPs and analysis of their manifestations at the level of gene expression are worthwhile tasks because many of such SNPs may be markers of clinical disorders.

During the “pregenomic era,” association of an SNP with a disease used to be a lucky finding [19–22], whereas, now, in the “postgenomic era,” identification of such associations is one of the goals of the 1000 Genomes Project [23]. Database dbSNP collects and ranks variants of each SNP by their prevalence [24]. The most frequent variant is entered into the reference human genome GRCh38 (NCBI) or hg38 (UCSC) (the terms used by the UCSC Genome Browser [25]) as an ancestral variant in the Ensembl database [26]. Minor alleles of SNPs in genes involved in a given pathological process can be found by means of the Web service* UCSC Genome Browser* [25], which visualizes a whole-genome map. Subsequent routine genotyping of these alleles in representative cohorts of patients and among healthy volunteers reveals (among minor alleles of SNPs) biomedical markers that are statistically significantly associated with the pathology in question [27]; this procedure takes up a lot of time and work. Computational (bioinformatic) analysis of many millions of unannotated SNPs from the 1000 Genomes Project may accelerate and cheapen the search for biomedical SNP markers.

Thus, the greatest success was achieved in the case of SNPs located in protein-coding regions of genes [28] because of the invariant (predictable) disruptions in the structure-function relations of the proteins encoded by these genes [29]. Moreover, advanced computer-based simulations of molecular dynamics and structures allowed researchers to predict in detail which SNPs would change the proteins. For example, molecular dynamics simulations provide deep analysis of the SNP-caused alterations in the amino acid arrangement that can affect the native three-dimensional atomic conformation of protein structure in order to estimate the most probable conformational modifications [30]. As an alternative/addition to molecular dynamics simulations for conformational sampling of proteins, so-called normal mode-based simulations guarantee multiscale modeling of protein conformational changes [31]. Besides, global minima of molecular docking for native and mutant structures can account for various substrate conformations and help identify an individual conformation with the most favorable binding energy [32]. In the case of drug resistance, computations of shape complementarity—between either widely used or promising new drugs and a binding pocket of a protein altered by an SNP—bring together the advantages of protein structure (or dynamics) simulations and the ability to dock one structure with another [33]. Finally, the alignment of multiple protein structures and/or sequences holds a key to the above calculations for comprehensive SNP analysis of protein-coding gene regions [34]. Meanwhile, the smallest progress was observed with regulatory SNPs because their manifestations may vary from cell to cell, from tissue to tissue, from patient to patient, and from subpopulation to subpopulation [26]. That is why computer-based prediction of candidate regulatory SNP markers of human diseases is a challenging problem for current functional genomics, genetics, and bioinformatics.

In our previous study [35], we described a freely available Web service, SNP_TATA_Comparator (created by us), and demonstrated its practical use on more than 40 biomedical SNP markers in the binding sites for TATA-binding protein (TBP) between positions −70 and −20 relative to the transcription start (the region where all such empirically proven sites are located [36, 37]). Recently, we showed suitability of this Web service for prediction of candidate SNP markers of complications of Mendelian diseases in obesity [38] and of autoimmune complications of these diseases [39] as well as SNP markers that can either enhance or weaken biological activity of oncogene inhibitors during cancer chemotherapy [40] (hereinafter, we use the term “Mendelian disease” according to the notation in database Online Mendelian Inheritance in Man, OMIM® [28]).

In the present work, we applied our Web service SNP_TATA_Comparator [35] to unannotated SNPs in binding sites of ТВР which are located near known SNP markers of Mendelian human diseases and, for this reason, can also cause the same pathologies if these SNPs change the affinity of ТВР for the same promoters of the same human genes. Furthermore, we found some data on biochemical markers of chronopathologies (where these markers have the effects on gene expression which are identical to the effects of the above SNPs) and clinical studies on the prevalence of these chronopathologies as complications of the Mendelian diseases caused by these SNPs. Finally, using SNP_TATA_Comparator [35], we analyzed all SNPs within 90 bp proximal promoter regions for all protein-coding transcripts of the genes of the circadian clock core. As a result, we identified 53 candidate SNP markers of human chronopathologies; validation of these markers in accordance with clinical standards may make these SNPs useful for predictive-preventive personalized medicine [41].

## 2. Methods

### 2.1. Web Service

**Figure 1:**
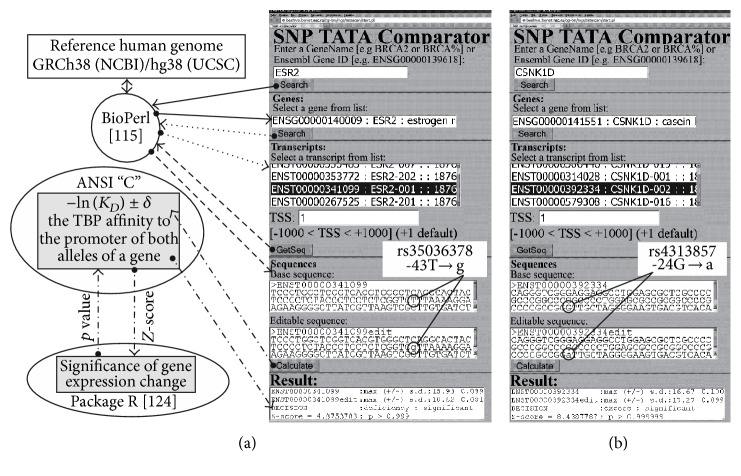
Examples of the predictions by SNP_TATA_Comparator [35] for statistically significant alterations in the affinity of TATA-binding protein for human gene promoters. (a) The known biomedical SNP marker rs35036378 located within a promoter of the human* ESR2 *gene associated with a Mendelian disease. This SNP is now predicted (in this study) to be a candidate SNP marker of circadian comorbidities and complications of diseases that one can see in Table 1. (b) The candidate SNP marker rs4313857 identified in this study within the human* CSNK1D* gene (belongs to the circadian clock core), as shown in Table 2.

Web service SNP_TATA_Comparator [35] is a bioinformatics application freely available on the Web (Figure 1; URL: http://beehive.bionet.nsc.ru/cgi-bin/mgs/tatascan/start.pl), which allows a user (i) to find an ancestral variant of the promoter for a transcript under study (the “Base sequence” text box) from the reference human genome (solid, dashed, dotted, and boldfaced arrows; BioPerl [115] is used), (ii) to introduce a mutation of interest (the “Editable sequence” text box), and (iii) to assess (the “Calculate” button) the values of ТВР's affinity for these two promoter variants, the relative mutation-related change in transcript levels, and statistical significance according to *Z*-score (the “Result” text box) as described in detail in our previous study [35].

### 2.2. The Bioinformatics Model

For each proximal 90 bp DNA sequence {*s*_−90_ ⋯ *s*_−1_} of a given gene promoter (where *s*_*i*_ ∈ {*a*, *c*, *g*, *t*}; *s*_0_ is the transcription start site), our Web service SNP_TATA_Comparator [35] calculates the maximal value of −ln⁡(*K*_*D*_) ± *δ* of the estimate of TBP's binding affinity for the 26 bp window {*s*_*i*−13_ ⋯ *s*_*i*_ ⋯ *s*_*i*+12_} [116, 117] (where −70 ≤ *i* ≤ −20 in both DNA strands; *K*_*D*_ is the equilibrium dissociation constant of the TBP-DNA complex, expressed in moles per liter; M), as follows:

(1) ln⁡KD=10.9−0.2ln⁡K1TA;μ+ln⁡K2PWMBucher+ln⁡K3WR;TV;

(2) δ=178·∑φ∈a,t,g,c ∑j=−1312ln⁡KDsi−13⋯si+j−1si+jsi+j+1⋯s−1KDsi−13⋯si+j−1φsi+j+1⋯s−1,

where 10.9 (natural logarithm units) is empirical nonspecific TBP-DNA affinity, 10^−5 ^M [118]; 0.2 is the stoichiometric coefficient; *K*_1_ is an empirical estimate of the equilibrium constant of TBP sliding along DNA; the average values of TBP's affinity for double-stranded DNA were estimated using the minor groove width (*μ*) and the TA dinucleotide content, [TA] [119]. *K*_2_ is an empirical estimate of the equilibrium constant of the primary corecognition between TBP and an appropriate TBP-biding site on DNA [−ln⁡(*K*_2_) is the maximal score of Bucher's position-weighted matrix: PWM_Bucher_] [120]. *K*_3_ is an empirical estimate of the equilibrium constant of stabilization of the TBP-DNA complex due to the bend of the axis of the DNA helix by an angle of 19° to 90° [121, 122] which depends on abundance of two TA-rich dinucleotides, WR ∈ {AA, AG, TA, TG} and TV ∈ {TA, TG, TC} [123]; *δ* is the standard deviation of *K*_*D*_ estimates for all the possible mononucleotide substitutions within the 26 bp DNA sliding window corresponding to the maximal *K*_*D*_ value found for the DNA sequence under study.

For two DNA sequences of the minor (mut) and ancestral (wt) alleles being compared, (1) and (2) yield {−ln⁡(*K*_*D*_^(mut)^) ± *δ*_(mut)_} and {−ln⁡(*K*_*D*_^(wt)^) ± *δ*_(wt)_}, respectively. Our Web service SNP_TATA_Comparator [35] compares them using Fisher's *Z*-score [124]:

(3) $$\begin{array}{c} Z=\frac{\left|\ln\left(K_{D}^{\left(mut\right)}/K_{D}^{\left(wt\right)}\right)\right|}{\sqrt{\delta_{\left(mut\right)}^{2}+\delta_{\left(wt\right)}^{2}}}. \end{array}$$

Using the standard statistical package R [124], we transform *Z*-score into *p* value of the probability of acceptance of H_0_ hypothesis “*K*_*D*_^(mut)^ ≠ *K*_*D*_^(wt)^” (where *α* = 1 − *p* is the statistical significance). Two cases, “*K*_*D*_^(mut)^ < *K*_*D*_^(wt)^” and “*K*_*D*_^(mut)^ > *K*_*D*_^(wt)^,” correspond, respectively, to overexpression and underexpression of the gene under study [125]. For more details, see our previous article [35].

### 2.3. Keyword Search

**Figure 2:**
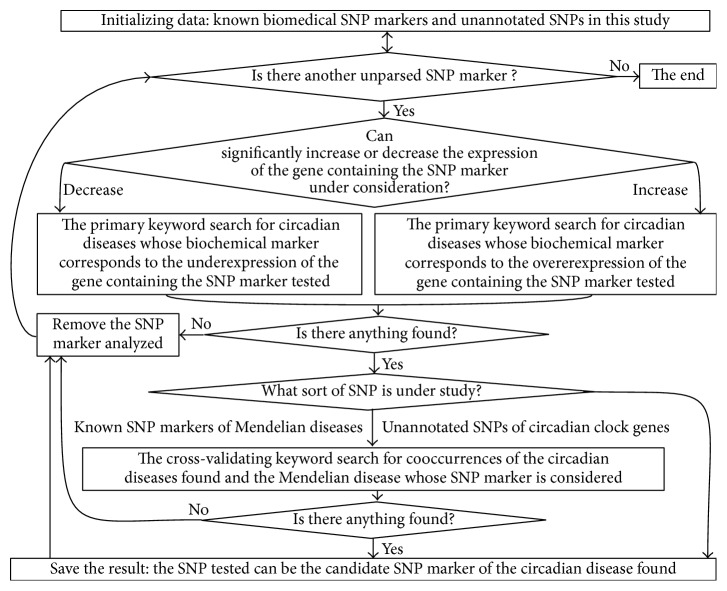
A flow chart showing our manual keyword search for chronopathologies (whose known biochemical markers correspond to our predictions of a significant change in the affinity of TATA-binding protein for human gene promoters).

Figure 2 shows this keyword search for data on known biochemical markers of chronopathologies; these markers correspond to predictions of SNP_TATA_Comparator (Figure 1) regarding a relative mutation-induced change in gene expression. For each known or candidate SNP marker causing either significant overexpression or underexpression of the human gene containing the SNP, we performed a manual keyword search using various combinations of the terms “overexpression,” “deficiency,” “circadian,” and many others corresponding to chronopathologies in public databases, as described in detail elsewhere [126]. In the case of genes of the circadian clock core, the obtained data are shown in Table 2 as results of this study. For SNP markers of Mendelian diseases, we conducted an additional keyword search for data on the prevalence of the uncovered chronopathologies as complications of these diseases; this procedure is some sort of cross-validation of the rough qualitative rates without statistical testing (Table 1).

Our heuristic interpretation of the keyword search results is shown in* italics* in the second rightmost column of Tables 1 and 2 and labeled with the word “*(Hypothetically)*” in front. We cite the studies (found during our manual keyword search) within the rightmost column of these tables, shown as [references] in* italics* and labeled with the phrase “*[This work]*.”

## 3. Results and Discussion 

### 3.1. The Results on Candidate SNP Markers of Circadian Complications of Mendelian Diseases

These results are presented in Table 1. Let us review in detail these more comprehensively studied SNP markers in order to briefly describe, in a similar fashion, the candidate SNP markers in the genes of human circadian clock core which were identified for the first time (in our study).

*Genes HBB and HBD* encode *β*- and *δ*-chains of hemoglobin, respectively. In the binding sites for TBP in their promoters, these two genes contain the greatest number (seven) of known SNP markers (rs35518301, rs397509430, rs33981098, rs34598529, rs33931746, rs33980857, and rs34500389) of thalassemia and resistance to malaria [24, 42], as a result of a hemoglobin deficiency (Table 1). A primary search by keywords uncovered a hemoglobin deficiency as a biochemical marker of circadian (nocturnal) aggravation of restless legs syndrome [43] and sensorineural hearing loss [44]. A cross-validating search by keywords revealed that iron deficiency anemia substantially contributes to the pathogenesis of restless legs syndrome and cooccurs with thalassemia [45, 46], whereas sensorineural hearing loss is a complication of thalassemia in children during treatment with deferoxamine [47]. We found three additional unannotated SNPs (rs63750953, rs281864525, and rs34166473) that can also reduce expression of genes* HBB* and* HBD* and may serve as candidate SNP markers of these chronopathologies.

*The MMP12 gene* codes for matrix metalloproteinase 12 and, in its promoter, contains a known SNP marker (rs2276109) of a lower risk of systemic sclerosis [48], psoriasis [49], and asthma [50]. A keyword search yielded circadian (nocturnal) aggravation of asthma symptoms [51]. Here we found an unannotated SNP (rs572527200) with the same effects on the ТВР-promoter affinity.

*Gene IL1B* encodes interleukin 1*β* and, in its promoter, contains one of the most widely studied SNP markers (rs1143627) of stomach ulcer, chronic gastritis, gastric cancer, hepatocellular carcinoma, non-small cell lung cancer, Graves' disease, and excess body fat in older men [52–57] as well as major depressive disorder [58] with a circadian optimum for diagnosis and treatment [59] that can be shifted by a high-fat or high-carbohydrate diet [60]. The primary search by keywords uncovered association of* IL1B* overexpression (with “-31T”) with a bipolar disorder [61] that also has a circadian optimum for diagnosis and treatment depending on the diet [60]. Near this known SNP marker, we found unannotated rs549858786, which was found to lower* IL1B* expression (Table 1). The primary keyword search produced an IL1B protein deficiency as a biochemical marker of rheumatoid arthritis [62], for which an additional keyword search yielded a study showing that this disease is associated with disturbances of the circadian rhythm of* IL1B* expression [63].

*The F3 gene *encodes tissue thromboplastin (factor III) and, in its promoter, contains a known SNP marker (rs563763767) of an elevated risk of venous thromboembolism and myocardial infarction [64]. A keyword search produced clinical data on circadian aggravation of their symptoms (in the early morning) in the elderly [65]; these data are in agreement with basic research on a murine model of aging [66].

*The F7 gene* codes for serum prothrombin conversion accelerator (factor VII); in its promoter, some researchers [67] found a biomedical SNP marker: a substitution of the ancestral nucleotide A for minor nucleotide C at position -33 relative to the transcription start site (hereafter -33A→С); this is a marker of moderate bleeding (as a result of underexpression of this gene). An additional database search revealed laboratory data on possible circadian aggravation of this disorder's symptoms during chronic changes of time zones and in the winter (data from a mouse model) [68]. Here we found an unannotated SNP (rs749691733) with the same effects on the ТВР-promoter interaction. In addition, near this known SNP marker, we found five unannotated SNPs (rs367732974, rs549591993, rs777947114, rs770113559, and rs754814507) that can cause* F7* overexpression (Table 1). A keyword search produced an elevated F7 protein level as a biochemical marker of heart attacks characterized by a circadian preference for the early morning in the elderly [69] and for circadian (postprandial) development of thrombogenesis [70]. Therefore, we propose rs367732974, rs549591993, rs777947114, rs770113559, and rs754814507 as candidate SNP markers of these two chronopathologies.

*Gene NOS2 *encodes inducible NO synthase; in its promoter, one study [71] uncovered an SNP marker (-51T→C) of resistance to malaria [71] and of a high risk of epilepsy [72] (as a result of overexpression of this gene). A keyword search yielded a review article [73] about epilepsy-associated hypothalamic damage that can impair the circadian clock system of the body as a whole [73]. Besides, we found some data [74] suggesting that excess NO is a biochemical marker of a remission of panic disorder that is characterized by circadian (late evening) aggravation of symptoms. Thus, we propose the SNP “NOS2: -51T→C” as a candidate marker of these chronopathologies.

*Gene DHFR* codes for dihydrofolate reductase; its promoter contains a known SNP marker (rs10168) of methotrexate resistance [75] that is characterized by a therapeutic optimum of its use [13]. Here we found an unannotated SNP (rs750793297) with the same effects on the ТВР-promoter complex. Additionally, near this known SNP marker, we found three unannotated SNPs (rs766799008, rs764508464, and rs754122321) that can cause DHFR underexpression (Table 1). According to our recent paper [40], these SNPs can elevate an apparent bioactivity of methotrexate-based antitumor chemotherapy [13, 75].

*The StAR gene* encodes steroidogenic acute regulatory protein and contains an SNP marker (rs16887226) of hypertension in diabetes (as a result of lowered expression of this gene because of impaired binding of its promoter with an unknown transcription factor, not TBP) [76]. A keyword search produced associations with lowered resistance to endotoxins for underexpression of the StAR protein, which is a mediator of mutual synchronicity of the immune system and circadian system [11]. Near this known SNP marker, we found the unannotated SNP rs544850971, which can lower* StAR* expression (Table 1) and therefore can be a candidate SNP marker of the above-mentioned disorders.

*Gene CETP* codes for cholesterol ester transfer protein; in its promoter, it contains a known biomedical SNP marker: deletion of the region G_−72_GGCGGACATACATATAC_−54_ (18 bp long) at position -54 relative to the transcription start site (hereafter: -54[18 bp]DEL); this is a marker of hyperalphalipoproteinemia that lowers the risk of atherosclerosis [77, 78]. A keyword search uncovered clinical data on circadian pathogenesis (postprandial flare-up) of this disorder in diabetes [79]. Near this known SNP marker, we found three unannotated SNPs (rs17231520, rs757176551, and rs569033466), which can increase* CETP* expression (Table 1) and thereby increase the risk of atherosclerosis [77–79] and of hypoalphalipoproteinemia which causes hepatic chronopathologies [80].

*The APOA1 gene* encodes apolipoprotein A1; in its promoter, some researchers [81] identified an SNP marker (-35A→С) of hematuria, hepatic steatosis, and obesity and of hypoalphalipoproteinemia which impairs the peripheral circadian clock in the liver [80]. A keyword search yielded some data on a knockout mouse model (APOA1^−/−^) regarding the risk of atherosclerosis [78] which develops in postprandial flare-ups in diabetes [79]. For this reason, we propose the SNP “APOA1: -31A→C” as a candidate marker of this chronopathology.

*Gene CYP2B6* encodes cytochrome P450 2B6 and contains a known SNP marker (rs34223104) of improved bioactivation of cyclophosphamide [82] with a circadian therapeutic optimum [14]. According to empirical and computational data [82], this SNP disrupts a major variant of the ТВР-binding site in the CYP2B6 promoter and in its place creates a binding site for the transcription factor (activator) C/EBP; this change shifts the ТВР-binding site and transcription start by 30 bp in the 5′ → 3′ direction and turns them into their minor alternative variants. In close proximity to this known SNP marker, we found the unannotated SNP rs563558831, which, in the same manner, lowers ТВР's affinity for this promoter (Table 1) and therefore can be a candidate SNP marker of the same chronopathology.

*The INS gene* encodes insulin, and its promoter contains a known SNP marker (rs5505) of neonatal diabetes and hyperinsulinemia [24]. A keyword search uncovered hyperinsulinemia as a biochemical marker of aberrations in the circadian rhythms of (i) the reproductive system [83], (ii) blood pressure [84], and (iii) the tumor-host balance [85]. Near this known SNP marker, we found unannotated rs563207167, which can also cause hyperinsulinemia and therefore can be a candidate SNP marker of the same chronopathologies (Table 1). In addition, here we found unannotated rs11557611, which can cause hypoinsulinemia (Table 1). A keyword search showed that hypoinsulinemia is a biochemical marker of hypothalamic amenorrhea [86]. Consequently, rs1155761 may serve as a candidate SNP marker of this chronopathology (Table 1).

*Gene ESR2* codes for estrogen receptor 2 (*β*) and, in its promoter, contains a known SNP marker (rs35036378) for prophylactic treatment (with tamoxifen) of an ESR2-deficient primary tumor pT1 [87] to prevent progression to breast cancer [88]; this treatment is characterized by a circadian optimum for its use [89]. A keyword search yielded basic research findings of circadian disturbances of daytime behavioral activity in ESR2-deficient female mice [90]. Near this known SNP marker, we found an unannotated SNP (rs35036378) with the same effects on the ТВР-promoter affinity.

### 3.2. The Results on Candidate SNP Markers within the Circadian Clock Core

These results are shown in Table 2. Let us review in more detail the data in this table using the* PER1 *gene as an example, which encodes a protein called period 1—a subunit of the heterodimeric PER-CRY complex—which is the main negative component of the circadian clock core: this complex inhibits the activity of transcription factor CLOCK/ARNTL [92–98].

As predicted by SNP_TATA_Comparator [35], only five of the 28 SNPs (that are known in the 90 bp proximal promoter regions for various protein-coding transcripts of this gene [24]) can affect the affinity of TBP for its promoters: rs137890200, rs773740924, and rs2518024 can enhance the TBP-promoter affinity, whereas rs796629786 and rs3027175 can reduce it. A keyword search showed that strong expression of the* PER1* gene inhibits the proliferation of tumor cells [17, 99, 100]; for example, in patients with strong expression of this gene, if they have a gastric cancer, longer survival is observed [99]. This gene is studied as a tumor suppressor; one of its mechanisms of action is the influence on the sensitivity of cells to DNA damage-induced apoptosis [17, 101]. Downregulation of PER1 was detected in human tissues of malignant tumors of the stomach and prostate [100, 101]. It should also be noted that, in studies of knockout mouse models (*PER1* ^−/−^), researchers observed impairment of spatial (3D) learning capacity and enhanced manifestations of ethanol hepatotoxicity [102, 103]. Therefore, we can hypothesize that rs137890200, rs773740924, rs2518024, rs796629786, and rs3027175 of the* PER1* gene are candidate SNP markers, as we propose in Table 2. One can see similar results for the other genes of the human circadian clock core [92–114] in this table.

Using SNP_TATA_Comparator [35], we analyzed 231 SNPs within 90 bp proximal promoter regions for the protein-coding transcripts of 12 genes of the human circadian clock core; only 52 of these SNPs (22%) were found to be capable of statistically significant changes in the affinity of TBP for promoters of these genes. As one can see in Table 2, we failed to find candidate SNP markers of chronopathologies for only one of the 12 genes, namely,* NR1D1*. This result shows that preliminary computational (bioinformatic) analysis of unannotated SNPs from the 1000 Genomes Project can indeed accelerate and cheapen the search for biomedical SNP markers because of selection (for this expensive and labor-intensive procedure) of only those candidate markers whose molecular mechanisms of pathological manifestation are easily understandable within the framework of existing clinical observations, genetic knowledge, scientific theories, hypotheses, and empirical data from animal and cellular models of human diseases.

It is also worth noting that only 13 of the 52 candidate SNP markers identified here decrease affinity of ТВР for promoters of the genes of the circadian clock core, whereas the other 39 SNPs enhance it. In Table 1, however, one can see the opposite distribution of the candidate SNP markers (identified here) of circadian complications of Mendelian diseases: the majority (26 of 41, 62%) of the candidate SNP markers significantly reduce affinity of ТВР for the human gene promoters, whereas the remaining 15 SNPs enhance it, as predicted by SNP_TATA_Comparator [35]. This difference is statistically significant (*p* < 0.0005) according to Fisher's exact test for 2 × 2 design. It is noteworthy (Table 1) that the ratio of the prevalence of candidate SNP markers of increased versus decreased affinity ТВР-promoter is in agreement with independent studies by other investigators [127, 128]. Indeed, overall, in the reference human genome, the proportion of SNPs which weaken the binding sites of transcription factors is significantly greater than the share of SNPs which enhance this binding [127]. Similarly, some researchers [128] reported that SNPs of the binding sites for transcription factor NF-*κ*B or RNA polymerase II (significantly more often) weaken rather than enhance the binding of these proteins to the mutated DNA in comparison with the reference genome. Taken together, these findings suggest that the reduced proportion of candidate SNP markers weakening the affinity TBP-promoter may be a specific characteristic of the 12 genes of the human circadian clock core. This phenomenon may reflect the pressure of natural selection for robustness of their functioning under the conditions of incessant genetic variability of the promoter region being analyzed.

Why is the robustness of the circadian clock core so important for humans? As shown in Table 2, overall, dysregulation of these genes' expression may be a marker of a wide range of pathological conditions in humans, for example, cancer, neurodegenerative disorders, lung diseases, and cardiovascular diseases. The reason for such diversity of chronopathologies is that the circadian clock synchronizes a large number of molecular biological and biochemical processes on the whole-body level and integrates various individual signals from each cell, tissue, and organ into a united hierarchical system of circadian rhythms of the human body.

### 3.3. How to Use Candidate SNP Markers of Chronopathologies

In this work, we used SNP_TATA_Comparator [35] to analyze 484 SNPs within 90 bp proximal promoter regions for protein-coding transcripts of human genes. Only 53 of these SNPs (11%) were found to be candidate SNP markers of chronopathologies (Tables 1 and 2). This finding does not mean that the remaining 431 of the 484 SNPs (89%) cannot be SNP markers of some human diseases. This is because each of these SNPs may influence a specific promoter-related nucleosome [129], DNA methylation sites in promoters, binding sites for histone modifications, and binding sites for transcription factors (e.g., rs16887226 and rs34223104). At present, there is a large number and variety of freely available Web services [130–149]. Most of them rank unannotated SNPs by their generalized statistical similarity with biomedical SNP markers of human diseases; these Web services evaluate this similarity by superimposing SNPs on gene maps and on data from massively parallel high-throughput sequencing of chromatin immunoprecipitation material (ChIP-Seq) from experiments with complexes of various proteins with genomic DNA. Accuracy of such assessments is constantly increasing due to improvements in empirical formulas for whole-genome evaluation of similarity among pathological manifestations of various SNPs and due to the increasing diversity, completeness, and number of whole-genome maps for various epigenetic states of cells from various tissues and organs in health [150], during infection [151] (or other diseases [152]), or after treatment [153], as we predicted [154] on the basis of the Central Limit Theorem.

As an unexpected clever generalization of this mainstream approach, the authors of Web server GenomeRunner [155] proposed to evaluate the difference between SNPs in addition to the widely accepted notion of assessments of the similarity between them. In this active field of research, the new trend is creation of Web navigation services that help users generate their own hypotheses and ideas regarding how the SNP of interest can affect the signs and symptoms of diseases under study [156]. Another innovation that emerged here is Web service PredictSNP2 for translation from the numerical predictions to an effect of an SNP on human health which is suitable for precise computer calculations in qualitative categories that are accessible to the general population [157]. These breakthroughs mean that SNP-related predictions are becoming interesting not only to narrow specialists who treat patients with one or another disease but also to anyone who is willing to customize their lifestyle to minimize the risk of diseases.

Because statistical significance of our predicted candidate SNP markers (Tables 1 and 2) varies from high (*α* < 10^−6^) to minimally acceptable (*α* < 0.05), the proposed markers should be properly validated using clinical standards before practical use. The results of this validation are dependent on climate, environmental conditions, and lifestyles and on the ethnic, social, age, and gender composition of cohorts [158]. Accordingly, we arranged the ancestral and minor alleles of each of candidate SNP markers of chronopathologies by the predicted *K*_*D*_ values of TBP-DNA affinity* in vitro* [91]. As shown in Tables 1 and 2, these *K*_*D*_ values vary from 1 to 335 nM, whereas the extent of their variation among alleles of a given SNP may be 1 nM, which is less than 0.3% of the *K*_*D*_ range. This level of allelic variations is too small for empirical measurement without an* a priori* known, fairly narrow range of *K*_*D*_ values to be measured. Thus, the predicted *K*_*D*_ values (Tables 1 and 2) are an integral part of each candidate SNP marker; without these data, an SNP marker cannot be validated in practice.

Finally, pathological manifestation of SNP markers of Mendelian diseases, as a rule, is limited to the consequences of changes in the expression of only those genes that contain these SNPs and can be useful only to physicians of the narrow specialties relevant to the diseases in question. Nonetheless, candidate SNP markers of chronopathologies are associated with consequences of desynchronoses either among the nervous, immune, digestive, respiratory, and other systems of the human body or between the human body and its environment (Tables 1 and 2). These data can be useful both for physicians and for the general population. For instance, the candidate SNP marker rs568650510 may be associated with an elevated risk of asthma whose symptoms are circadian (worse at night [51]; Table 2). Using this information, a physician can select the treatment timing (for asthma symptoms in a patient with minor alleles of these SNPs) that could reduce the risk of aggravation at night. By the same token, any person with the minor allele -15T of this SNP can choose a lifestyle that can reduce the systematic nocturnal influence of the environmental factor that causes the asthma symptoms. Similarly, rs367732974, rs549591993, rs192518038, and rs537333415 may help reduce the risk of a heart attack [69]; rs374778785 may be useful for lowering the risk of emphysema and lung cancer among smokers [106], whereas rs2899302 may help decide whether to use opioids [113].

## 4. Conclusions

Here, we predicted candidate SNP markers of chronopathologies (Tables 1 and 2); these SNPs can change affinity of TATA-binding protein for human gene promoters. After proper validation of these candidate markers in accordance with clinical standards, these SNPs may turn out to be useful both for physicians (to select the best treatment for each patient) and for the general population (to choose a lifestyle preventing possible circadian comorbidities and complications).

## Figures and Tables

**Table 1:** Candidate SNP markers of circadian complications and/or comorbidities of Mendelian diseases as predicted by a significant change in the affinity of TATA-binding protein for human gene promoters.

*Gene*	dbSNP [24] rel. 146					*K*_*D*_, nM		*Z*	*α*	Known Mendelian diseases (see [references]) *and (hypothetically) circadian complications and comorbidities whose candidate SNP markers were predicted by us in [this work] (see Figure 2)*	[References] or *[this work]* *(see Figure 2)*
	ID or [reference]	SNP **wt**→*mut*	5′ flank	**wt** *mut*	3′ flank	**wt** *mut*	Δ				
*HBB*	rs397509430	**-29t** *DEL*	gggctgggca	**t** *—*	atacaacagt	**5** *29*	↓	34	10^−6^	Malaria resistance and *β*-thalassemia *and (hypothetically) circadian symptoms (worse at night) in restless legs syndrome caused by iron deficiency anemia cooccurring with thalassemia and for sensorineural hearing loss as a complication of deferoxamine-based therapy in thalassemia *	[24, 42], *[this work]* *[43–47]*
	rs33980857	**-29t**→*a, g, c*	gggctgggca	**t** *a, g, c*	atacaacagt	**5** *21*	↓	27	10^−6^		
	rs34598529	**-28a**→*g*	ggctgggcat	**a** *g*	aaagtcaggg	**5** *18*	↓	24	10^−6^		
	rs33931746	**-27a**→*g, c*	gctgggcata	**a** *g, c*	aagtcagggc	**5** *11*	↓	14	10^−6^		
	rs33981098	**-30a**→*g, c*	agggctgggc	**a** *g, c*	taaaagtcag	**5** *9*	↓	10	10^−6^		
	rs34500389	**-31c**→*a, t, g*	cagggctggg	**c** *a, t, g*	ataaaagtca	**5** *6*	↓	3	10^−2^		

*HBB*	rs63750953	**-25aa** *DEL*	ctgggcataa	**aa** —	gtcagggcag	**5** *8*	↓	*9*	10^−6^	*(Hypothetically)malaria resistance, β-thalassemia, and circadian symptoms (worse at night) in restless legs syndrome and sensorineural hearing loss (for details, see above)*	*[This work]* *[42–47]*
	rs281864525	**-25a**→*c*	tgggcataaa	**a** *c*	gtcagggcag	**5** *7*	↓	*7*	10^−6^		

*HBD*	rs35518301	**-31a**→*g*	caggaccagc	**a** *g*	taaaaggcag	**4** *8*	↓	11	10^−6^	Malaria resistance and *δ*-thalassemia *and (hypothetically) circadian symptoms (worse at night) for restless legs syndrome and sensorineural hearing loss (for details, see above)*	[24, 42], *[this work]* *[43–47]*

*HBD*	rs34166473	**-30t**→*c*	aggaccagca	**t** *c*	aaaaggcagg	**4** *8*	↓	*18*	10^−6^	*(Hypothetically) malaria resistance, δ-thalassemia, and circadian symptoms (worse at night) for restless legs syndrome and sensorineural hearing loss (for details, see above)*	*[This work]* *[42–47]*

*MMP12*	rs2276109	**-27a**→*g*	gatatcaact	**a** *g*	tgagtcactc	**11** *14*	↓	3	10^−2^	Low risk of systemic sclerosis, psoriasis, and asthma whose symptoms are circadian (worse at night)	[48–51]

*MMP12*	rs572527200	**-30a** *→g*	gatgatatca	**a** *g*	ctatgagtca	**11** *14*	↓	*3*	10^−2^	*(Hypothetically) low risk of systemic sclerosis, psoriasis, and asthma whose symptoms are circadian (worse at night)*	*[This work]* *[48–51]*

*IL1B*	rs1143627	**-31c**→*t*	ttttgaaagc	**c** *t*	ataaaaacag	**5** *2*	↑	15	10^−6^	Gastric cancer in *Helicobacter pylori* infection, hepatocellular carcinoma in hepatitis C virus infection, non-small cell lung cancer, chronic gastritis and gastric ulcer in* H. pylori* infection, Graves' disease, greater body fat in older men, recurrent major depressive disorder whose diagnosis and therapy are characterized by circadian optima for use, *and (hypothetically) bipolar disorder whose diagnosis and therapy are characterized by circadian optimafor usedepending on the diet*	[24, 52–59], *[this work]* *[60, 61]*

*IL1B*	*rs549858786*	**-28a**→*t*	tgaaagccat	**a** *t*	aaaacagcga	**5** *6*	↓	*8*	10^−6^	*(Hypothetically) rheumatoid arthritis that can disrupt the circadian rhythm of IL1B gene expression*	*[This work]* *[62, 63]*

*F3*	rs563763767	**-21c**→*t*	ccctttatag	**c** *t*	gcgcggggca	**3** *2*	↑	6	10^−6^	Venous thromboembolism and myocardial infarction that are characterized by their circadian preference for the early morning in the elderly	[64–66]

*F7*	See [67]	**-33a**→*c*	ccttggaggc	**a** *c*	gagaactttg	**53** *62*	↓	3	10^−2^	Moderate bleeding tendency whose symptoms are circadian: worse with chronic change of time zones and in winter	[67, 68]

*F7*	rs749691733	**-21c**→*t*	agaactttgc	**c** *t*	cgtcagtccc	**53** *66*	↓	4	10^−3^	*(Hypothetically) moderate bleeding tendency whose symptoms are circadian: worse with chronic change of time zones and in winter*	*[This work]* *[67, 68] *

*F7*	rs367732974	**-19g**→*a*	aactttgccc	**g** *a*	tcagtcccat	**53** *47*	↑	*2*	*0.05*	*(Hypothetically) heart attacks that are characterized by circadian preference for the early morning in the elderly and circadian (postprandial) flare-ups of thrombogenesis*	*[This work]* *[69, 70]*
	rs549591993	**-13c**→*a*	gcccgtcagt	**c** *a*	ccatggggaa	**53** *25*	↑	*13*	10^−6^		
	rs777947114	**-23g**→*a*	agagaacttt	**g** *a*	cccgtcagtc	**53** *19*	↑	*19*	10^−6^		
	rs770113559	**-38g**→*a*	gtcacccttg	**g** *a*	aggcagagaa	**53** *41*	↑	*5*	10^−6^		
	rs754814507	**-54c**→*t*	cctcccccat	**c** *t*	cctctgtcac	**53** *45*	↑	*3*	10^−3^		

*NOS2*	See [71]	**-51t**→*c*	gtataaatac	**t** *c*	tcttggctgc	**2** *1*	↑	3	10^−2^	Resistance to malaria and high risk of epilepsy that damages the hypothalamus and the circadian clock as a whole *and (hypothetically) remission of panic disorder whose symptoms are circadian (worse late in the evening)*	[71–73], *[this work]* *[74]*

*DHFR*	rs10168	**-26g**→*a*	ctgcacaaat	**g** *a*	gggacgaggg	**15** *9*	↑	9	10^−6^	In leukemia, resistance to methotrexate therapy that is characterized by a circadian optimum for use	*[13, 75]*

*DHFR*	rs750793297	**-25g**→*t*	tgcacaaatg	**g** *t*	ggacgagggg	**15** *13*	↑	3	0.01	*(Hypothetically) in leukemia, resistance to methotrexate therapy that is characterized by a circadian optimum for use*	*[This work]* *[13, 75]*

*DHFR*	rs766799008	**-28a**→*g*	ctgcacaaat	**a** *g*	tggggacgag	**15** *19*	↓	3	10^−3^	*(Hypothetically) enhancement of an apparent bioactivity of methotrexate therapy that may be characterized by a circadian optimum for use*	*[This work]* *[13, 40, 75]*
	rs764508464	**-a28*DEL***	ctgcacaaat	**a** *—*	tggggacgag	**15** *37*	↓	17	10^−6^		
	rs754122321	**-31c**→*g*	ctcgcctgca	**c** *g*	aaatggggac	**15** *25*	↓	9	10^−3^		

*StAR*	rs16887226	**-33c**→*t*	cagccttcag	**c** *t*	gggggacatt	**10** *10*	=	0	>0.5	Hypertension in diabetes (EMSA: an unknown TF-binding site is disrupted rather than a TBP-binding site) and *(hypothetically) weak circadian resistance to exotoxins (deficiency of the mediator between the circadian and immune systems)*	[76] *[this work]* *[11]*

*StAR*	rs544850971	**-22a**→*g*	tcagcggggg	**a** *g*	catttaagac	**10** *12*	↓	*5*	10^−2^	*(Hypothetically) hypertension in diabetes and weak circadian resistance to exotoxins*	*[This work]* *[11, 76]*

*CETP*	See [77]	**-54**[**18 bp**]*DEL*	cgtgggggct	**[18]** —	gggctccagg	**4** *7*	↓	7	10^−6^	Hyperalphalipoproteinemia reduces the risk of atherosclerosis that is characterized by circadian (postprandial) flare-ups	[77–79]

*CETP*	rs17231520	**-68g**→*a*	ggggctgggc	**g** *a*	gacatacata	**4** *2*	↑	*10*	10^−6^	*(Hypothetically) risk of atherosclerosis that is characterized by circadian (postprandial) flare-ups and hypoalphalipoproteinemia causing chronopathologies of the liver*	*[This work]* *[80]*
	rs569033466	**-53g**→*a*	atacatatac	**g** *a*	ggctccaggc	**4** *3*	↑	*4*	10^−3^		
	rs757176551	**-49c**→*g*	catatacggg	**c** *g*	tccaggctga	**4** *2*	↑	*10*	10^63^		

*APOA1*	See [81]	**-35a**→*c*	tgcagacata	**a** *c*	ataggccctg	**3** *4*	↓	5	10^−3^	Hypoalphalipoproteinemia causing chronopathologies in the liver, as well as hematuria, fatty liver, and obesity, *and (hypothetically) atherosclerosis that is characterized by circadian (postprandial) flare-ups*	[80, 81] *[this work]* *[77–80]*

*CYP2B6*	rs34223104	**-28t**→*c*	gatgaaattt	**t** *c*	ataacagggt	**4** *10*	↓	15	10^−6^	Better metabolic activation of anticancer prodrug cyclophosphamide that is characterized by a circadian optimum for use	[14, 82]

*CYP2B6*	rs563558831	**-26t**→*c*	tgaaatttta	**t** *c*	aacagggtgc	**4** *10*	↓	*13*	10^−6^	*(Hypothetically) better metabolic activation of cyclophosphamide that is characterized by a circadian optimum for use*	*[This work]* *[14, 82] *

*INS*	rs5505	**-9c**→*t*	agatcactgt	**c** *t*	cttctgccat	**53** *44*	↑	4	10^−6^	Type 1 diabetes after neonatal diabetes mellitus and risk of hyperinsulinemia that disturbs circadian rhythms of the reproductive system, of blood pressure, and of tumor-host balance	[24, 83–85]

*INS*	rs563207167	**-28c**→*t*	tcagccctgc	**c** *t*	tgtctcccag	**53** *44*	↑	4	10^−3^	*(Hypothetically) type 1 diabetes after neonatal diabetes mellitus and risk of hyperinsulinemia that disturbs circadian rhythms of the reproductive system, of blood pressure, and of tumor-host balance*	*[This work]* * [24, 83–85] *

*INS*	rs11557611	**-8c**→*t*	gatcactgtc	**c** *t*	ttctgccatg	**53** *60*	↓	2	0.05	*(Hypothetically) hypothalamic amenorrhea*	*[This work]* *[86]*

*ESR2*	rs35036378 (see Figure 1(a))	**-43t**→*g*	cctctcggtc	**t** *g*	ttaaaaggaa	**6** *8*	↓	5	10^−3^	Preventive therapy for an ESR2-deficient pT1 primary tumor against its progression to breast cancer by means of tamoxifen when this treatment is characterized by a circadian optimum for use *and (hypothetically) disturbances in circadian (daytime) behavioral activity*	[87–89] *[this work]* *[90]*

*ESR2*	rs766797386	**-32g**→*t*	ttaaaaggaa	**g** *t*	aaggggctta	**6** *7*	↓	3	10^−2^	*(Hypothetically) preventive therapy for an ESR2-deficient primary pT1 tumor against its progression to breast cancer by means of tamoxifen when this treatment is characterized by a circadian optimum for use; disturbances in circadian (daytime) behavioral activity*	*[This work]* *[87–90]*

wt, ancestral allele; mut, minor allele; *K*_*D*_, an estimate [35] of the dissociation constant (*K*_*D*_) of the TBP-DNA complex *in vitro* [91]; Δ, a gene expression change in comparison with the norm (=): overexpression (↑) and underexpression (↓); *Z*, *Z*-score; *α* = 1 − *p*, significance (where *p* is a probability rate of acceptance of H_0_ hypothesis “*K*_*D*_^(mut)^  ≠  *K*_*D*_^(wt)^”; see Figure 1); TF, transcription factor; EMSA, electrophoretic mobility shift assay.

**Table 2:** Candidate SNP markers within the human genes of the circadian clock core as predicted by a significant change in the affinity of TATA-binding protein for human gene promoters.

*Gene*	dbSNP [24] rel. 146					*K*_*D*_, nM		*Z*	*α*	*Circadian complications and comorbidities whose candidate SNP markers were predicted in [this work] (clinical data, laboratory animals, or cellular model)*	*[References] found (see Figure 2)*
	ID	SNP **wt**→*mut*	5′ flank	**wt** *mut*	3′ flank	**wt** *mut*	Δ				
*CLOCK*	rs192518038	**-57g**→*t*	aggacctaag	**g** *t*	ctagcgctct	**63** *29*	↑	14	10^−6^	*(Hypothetically) higher risk of heart attacks that are characterized by circadian preference for the early morning in the elderly with diabetes (CLOCK-mutant mice)*	*[This work]* *[92]*
	rs537333415	**-63c**→*t*	gcctccagga	**c** *t*	ctaaggctag	**63** *45*	↑	7	10^−6^		

*ARNTL*	rs534789405	**-28g**→*a*	cggattggct	**g** *a*	ggggcggccg	**184** *89*	↑	12	10^−6^	*(Hypothetically) malignant pleural mesothelioma (13 malignant pleural mesothelioma cell lines in comparison with the nontumorigenic mesothelial cell line MeT-5A)*	*[This work]* *[93]*
	rs758737644	**-39c**→*t*	tgcactgtta	**c** *t*	acattctgtt	**10** *3*	↑	4	10^−3^		
	rs549031146	**-46t**→*a*	caaaacttat	**t** *a*	gggtgctatg	**6** *5*	↑	4	10^−3^		

*ARNTL2*	rs776246315	**-21g**→*a, (t)*	ttccagccgc	**g** *a, (t)*	tgagtccagg	**49** *31*	↑	8	10^−6^	*(Hypothetically) inhibition of type 1 diabetes (murine model)*	*[This work]* *[94]*
	rs140915764	**-22c**→*t*	tttccagccg	**c** *t*	gtgagtccag	**49** *32*	↑	7	10^−6^		
	rs756988598	**-23g**→*a*	gtttccagcc	**g** *a*	cgtgagtcca	**49** *39*	↑	4	10^−3^		
	rs753093730	**-33g**→*a, t*	ctgcccatag	**g** *a, t*	taaagtgttg	**7** *5*	↑	3	10^−3^		
	rs536395877	**-46c**→*a*	ttgttgtact	**c** *a*	tgctgcccat	**7** *5*	↑	5	10^−3^		
	rs769981079	**-42g**→*a, t*	ccagtgcatt	**g** *a, t*	ctcctgtggt	**49** *26*	↑	12	10^−6^		
	rs111899732	**-46c**→*t*	agaaccagtg	**c** *t*	attgctcctg	**49** *14*	↑	18	10^−6^		
	rs746050396	**-57g**→*a*	gttgagagag	**g** *a*	agaaccagtg	**49** *36*	↑	5	10^−6^		

*ARNTL2*	rs369143719	**-48c**→*t*	tcttgttgta	**c** *t*	tctgctgccc	**7** *9*	↓	4	10^−3^	*(Hypothetically) suppression of diabetes protection (murine model)*	*[This work]* * [95]*
	rs374142420	**-51g**→*c*	atgtcttgtt	**g** *c*	tactctgctg	**7** *10*	↓	4	10^−3^		
	rs770635249	**-56[ttg]** *DEL*	aataaatgtc	**ttg** —	ttgtactctg	**7** *10*	↓	4	10^−3^		

*CRY1*	rs747100146	*INS-49tt*	gataggagtt	**—** *tt*	aattatccta	**6** *7*	↓	2	0.05	*(Hypothetically) susceptibility to arthritis (CRY1* ^−/−^ *CRY2* ^−/−^ * knockout mice)*	*[This work]* * [96]*

*CRY2*	rs753656899	**-16a**→*g*	gcggggacta	**a** *g*	gggtggagtt	**27** *56*	↓	12	10^−6^	*(Hypothetically) susceptibility to arthritis and to mood disorders (CRY1* ^−/−^ *CRY2* ^−/−^ * and CRY2* ^−/−^ * knockout mice, resp.)*	*[This work]* *[96, 97] *

*CRY2*	rs575588903	**-9a**→*g*	ctaagggtgg	**a** *g*	gttgcggcgt	**27** *25*	↑	2	0.05	*(Hypothetically) resistance to chemotherapy and poor prognosis in colorectal cancer (colorectal cancer samples)*	*[This work]* *[98]*
	rs757256843	**-14g**→*t*	ggggactaag	**g** *t*	gtggagttgc	**27** *13*	↑	10	10^−6^		
	rs760179689	**-24g**→*a*	ccctgtgggc	**g** *a*	gggactaagg	**27** *22*	↑	1	10^−3^		
	rs529410313	**-47c**→*a*	agctgtcagt	**c** *a*	ttgcaagtca	**22** *18*	↑	3	10^−2^		

*PER1*	rs137890200	**-17c**→*t*	gccaataagg	**c** *t*	ggagagtgtg	**21** *14*	↑	6	10^−6^	*(Hypothetically) longer survival among patients with gastric cancer*	*[This work]* *[99]*
	rs773740924	**-26c**→*a*	ctcgccctgg	**c** *a*	caataaggcg	**21** *14*	↑	7	10^−6^		
	rs2518024	**-60g**→*a*	gtgctctgga	**g** *a*	ttaaaccagc	**17** *8*	↑	12	10^−6^		

*PER1*	rs796629786	**-13t**→*g*	tcggcgcccc	**t** *g*	aagccaataa	**56** *118*	↓	13	10^−6^	*(Hypothetically) prostate cancer; ethanol hepatotoxicity, defects in hippocampal development, and resulting impairment of spatial learning capacity (PER1* ^−/−^ * knockout mice)*	*[This work]* *[17, 100–103]*
	rs3027175	**-67c**→*t*	ccagcaggtg	**c** *t*	tctggagtta	**17** *19*	↓	2	0.05		

*PER2*	rs780846747	**-20c**→*t*	cacaccttgt	**c** *t*	aagtagaaga	**10** *5*	↑	12	10^−6^	*(Hypothetically) susceptibility to acute Q fever in male patients; suppression of tumor growth (cell line S-180)*	*[This work]* *[104, 105]*

*RORA*	rs750596430	**-13c**→*t*	agccaggcag	**c** *t*	agcggcgcgg	**106** *60*	↑	10	10^−6^	*(Hypothetically) emphysema and its progression to lung cancer among smokers (mice, smoking machine TE-10)*	*[This work]* *[106]*
	rs535050458	**-20c**→*t*	gccagcgagc	**c** *t*	aggcagcagc	**106** *72*	↑	7	10^−6^		
	rs547170533	**-21c**→*a*, *(t)*	cgccagcgag	**c** *a, (t)*	caggcagcag	**106** *72*	↑	7	10^−6^		
	rs551503425	**-50c**→*t*	tgccaatatc	**c** *t*	aagggttgcc	**19** *6*	↑	15	10^−6^		
	rs374778785	**-56a**→*t*	attatccccc	**a** *t*	tactcctccc	**34** *28*	↑	4	10^−3^		

*RORA*	rs764749271	**-28[12 bp]** *DEL*	ccttctcctt	**[12 bp]** —	tttttttttt	**28** *31*	↓	2	0.05	*(Hypothetically) severe cerebellar ataxia (RORA* ^−/−^ * knockout mice)*	*[This work]* *[107]*
	rs762440045	**-55a**→*c*	acatggagtc	**a** *c*	gctccggcag	**106** *245*	↓	15	10^−6^		

*RORC*	rs568650510	**-15c**→*t*	actccttttc	**c** *t*	ctgcctgctg	**55** *25*	↑	14	10^−6^	*(Hypothetically) neurological manifestations of Behçet syndrome and asthma whose symptoms are circadian (worse at night) (18 patients and 30 pediatric patients, resp.)*	*[This work]* *[51, 108, 109]*

*CSNK1E*	rs777965060	**-12c**→*t*	aggccctctg	**c** *t*	ttgaccccca	**80** *69*	↑	3	0.05	*(Hypothetically) higher risk of c-MYC-dependent carcinogenesis, testicular cancer, and overproduction of cerebral β-amyloid*	*[This work]* *[110–112]*
	rs369188273	**-16c**→*t*	ccctcccctc	**c** *t*	gcgcccgctc	**288** *195*	↑	7	10^−6^		
	rs558609213	**-18c**→*t*	tcttttcttg	**c** *t*	atccctgcag	**30** *9*	↑	21	10^−6^		
	rs201914168	**-20g**→*t*	cctgacagag	**g** *t*	ccctctgctt	**80** *53*	↑	7	10^−6^		
	rs374953337	**-25c**→*t*	tgacccctga	**c** *t*	agaggccctc	**80** *27*	↑	17	10^−6^		
	rs368019196	**-31c**→*t*	tgcctctgac	**c** *t*	cctgacagag	**80** *68*	↑	3	10^−2^		
	rs775747363	**-40[gcc]** *DEL*	ctggctgcct	**gcc** —	tctgacccct	**80** *67*	↑	4	10^−3^		
	rs780432736	**-51g**→*a*	gccgggcgtg	**g** *a*	ctggctgcct	**80** *71*	↑	2	0.05		
	rs747636670	**-52g**→*a*	ggccgggcgt	**g** *a*	gctggctgcc	**80** *56*	↑	5	10^−6^		

*CSNK1E*	rs775283367	**-11c**→*g*	ctcttaccta	**c** *g*	gtcagctctt	**8** *10*	↓	4	10^−3^	*(Hypothetically) increased responsiveness to opioids (CSNK1E* ^−/−^ * knockout mice)*	*[This work]* *[113]*
	rs746761879	**-18t**→*c*	ccgactactc	**t** *c*	tacctacgtc	**8** *11*	↓	5	10^−6^		
	rs777083641	**-22[ag]** *DEL*	ctggctgcct	**ag** —	tctgacccct	**80** *93*	↓	3	10^−2^		
	rs2899302	**-59g**→*c*	cgagaaaact	**g** *c*	cgcgaggcct	**288** *335*	↓	3	10^−2^		

*CSNK1D*	rs4313857 (see Figure 1(b))	**-24g**→*a*	gccccgccgg	**g** *a*	ttgctagggg	**57** *32*	↑	8	10^−6^	*(Hypothetically) breast cancers (27 surgically resected tumor samples)*	*[This work]* *[114]*
	rs571866458	**-38c**→*a*	gagccggacc	**c** *a*	gcagtagcgg	**54** *42*	↑	4	10^−3^		
	rs540139460	**-56g**→*a*	gcagggtcgg	**g** *a*	aggaggcctg	**253** *145*	↑	10	10^−6^		
